# Complete genome sequence of *Helicobacter pylori* B128 7.13 and a single‐step method for the generation of unmarked mutations

**DOI:** 10.1111/hel.12587

**Published:** 2019-05-07

**Authors:** Emma M. Dawson, Karl A. Dunne, Emily J. Richardson, Judyta Praszkier, Dana Alfawaz, Simon Woelfel, Amanda De Paoli, Hassan Chaudhry, Ian R. Henderson, Richard L. Ferrero, Amanda E. Rossiter

**Affiliations:** ^1^ Institute of Microbiology and Infection College of Medical and Dental Sciences University of Birmingham Birmingham UK; ^2^ Hudson Institute for Medical Research, Monash Melbourne Victoria Australia; ^3^ Infection and Immunity Program, Monash Biomedicine Discovery Institute and Department of Microbiology Monash University Melbourne Victoria Australia

**Keywords:** gene mutation, genetic, *Helicobacter pylori*

## Abstract

**Background:**

*Helicobacter pylori* represents an interesting model of bacterial pathogenesis given that most infections are asymptomatic, while a minority of infections cause severe gastric disease. *H pylori* strain B128 7.13 is used extensively to understand *H pylori* pathophysiology. Due to extensive restriction‐modification systems, the fact that only some *H pylori* strains are naturally transformable, the inability of common plasmid and transposon vectors to replicate in this bacterium, as well as the limited number of antibiotic cassettes that are functional in *H pylori*, there are relatively few genetic tools for the mutagenesis of this bacterium.

**Materials and Methods:**

Here, we use PacBio and Illumina sequencing to reveal the complete genome sequence of *H pylori* B128 7.13. Furthermore, we describe a system to generate markerless and scarless mutations on the *H pylori* chromosome using the counter‐selection marker, galactokinase from *Escherichia coli*.

**Results:**

We show that this mutagenesis strategy can be used to generate in‐frame insertions, gene deletions, and multiple independent mutations in B128 7.13. Using the closed genome as a reference, we also report the absence of second site chromosomal mutations and/or rearrangements in our mutagenized strains. We compare the genome sequence of *H pylori* B128 7.13 with a closely related strain, *H pylori* B8, and reveal one notable region of difference, which is a 1430 bp insertion encoding a *H pylori‐*specific DUF874 family protein of unknown function.

**Conclusions:**

This article reports the closed genome of the important *H pylori* B128 7.13 strain and a mutagenesis method that can be adopted by researchers as an alternative strategy to generate isogenic mutants of *H pylori* in order to further our understanding of this bacterium.

## 1 INTRODUCTION

In contrast to *Escherichia coli, Helicobacter pylori* supports efficient double recombination in the absence of an exogenous recombinase^1^ and some strains are naturally competent for the uptake of DNA. However, due to the presence of multiple restriction‐modification systems^2^ and the inability of *H pylori* to support the replication of common plasmid and transposon vectors, there is a need to expand the toolbox for the mutagenesis of *H pylori.* Although many *H pylori* genes have been characterized by cloning and expression in laboratory *E coli* strains, expression of some *H pylori* proteins can be deleterious to *E coli* 3, 4 and so prevents their characterization. Traditional methods for *H pylori* mutagenesis often involve multiple cloning steps, which precede transformation with donor DNA to generate mutants marked with an antibiotic resistance cassette at the target loci.^5^ However, integration of an antibiotic resistance cassette introduces pitfalls to subsequent phenotypic analysis of isolated mutants as antibiotic resistance determinants can introduce polar effects on downstream gene expression.^6^ Furthermore, these conventional approaches limit the ability to introduce multiple mutations, due to the lack of available antibiotic resistance cassettes for use in *H pylori.* Thus, counter‐selectable markers emerged as a powerful tool that enabled negative selection for the removal of gene products, whose expression is toxic in the presence of certain substrates. Counter‐selection methods are now widely accepted as a superior mutagenesis strategy to generate markerless and scarless mutations in various important pathogens.^7^, ^8^

Previously, Copass et al developed a sucrose‐based counter‐selection method that utilized the kanamycin resistance cassette and the *sacB* gene from *Bacillus subtilis* for the introduction of unmarked mutations in *H pylori* strain G27.^10^ Although this counter‐selection method has been successfully implemented to generate unmarked mutations in *H pylori*,^2^ high frequency spontaneous mutations that give rise to sucrose resistance have been reported by multiple groups, ultimately hindering the use of this system.^11^ A counter‐selectable marker based on streptomycin susceptibility was also developed for *H pylori* mutagenesis, yet this requires streptomycin resistance in the host strain, cloning of two mutagenic plasmids and a two‐step transformation method.^4^, ^11^ To overcome these extensive cloning steps, Debowski et al, developed a mutagenic system combining streptomycin susceptibility with the difH/XerH excision and recombination system in *H pylori* to generate markerless gene deletions in a single transformation step.^12^ However, XerH‐mediated excision of the mutagenic cassette resulted in a 40‐bp scar region at the target site, limiting its effectiveness in generating in‐frame mutations within protein‐coding sequences. Therefore, there remains a need to develop alternative mutagenesis strategies to optimize the generation of markerless and scarless isogenic mutants.

Here, we describe an improved counter‐selection mutagenesis strategy in the *H pylori* strain B128 7.13, which requires only one cloning step and a single transformation to generate the desired markerless and scarless mutant strains. To do this, we used the counter‐selectable marker *galactokinase* (*galK*) gene from *E coli.* This gene has been used to generate markerless mutations in bacteria, such as *Mycobacterium smegmatis and Mycobacterium tuberculosis*.^7^ galK encodes galactokinase, an enzyme responsible for catalyzing the phosphorylation of galactose to galactose‐1‐phosphate. Additionally, GalK can also phosphorylate the galactose analogue, 2‐deoxy‐galactose (2‐DOG), leading to the production of 2‐deoxy‐galactose‐1‐phosphate, which cannot be further metabolized by *H pylori* leading to a buildup of toxic levels and subsequent cell death.^7^ We show that GalK expression in *H pylori* confers sensitivity to 2‐deoxy‐galactose (2‐DOG)*.* Thus, we synthesized a mutagenesis gene module comprising a codon‐optimized antibiotic resistance cassette, encoding either kanamycin or apramycin resistance, and the *galK* gene under the control of the strong *H pylori flaA* flagellin promoter.^13^ Using *H pylori* strain B128 7.13, we have shown that this counter‐selection method can be used to introduce multiple genomic modifications, such as the insertion of in‐frame epitopes and deletion of target genes. Moreover, we show that this strategy can be used to manipulate the *H pylori* genome at multiple loci by consecutive rounds of mutagenesis, avoiding the conventional approach of introducing multiple antibiotic resistance markers. Lastly, we reveal the first fully assembled whole genome sequence of *H pylori* strain B128 7.13, enabling genome‐wide analysis of our mutant strains, which confirms the presence of our markerless and scarless mutations in the absence of second site mutations.

## 2 METHODS

### 2.1 Bacterial strains and media

*Helicobacter pylori* B128 7.13 (*cag*PAI^+^/T4SS^+^) is a mouse‐colonizing strain that has been described previously.^14^ *H pylori* strains were routinely cultured on either horse blood agar (HBA) or in brain heart infusion broth (BHI; Oxoid) containing a modified Skirrow's selective supplement (comprising 10 µg/mL vancomycin, 25 ng/mL polymyxin B, 5 µg/mL trimethoprim, and 2.5 μg/mL amphotericin B) according to standard procedures.^15^ Medium was supplemented with 25 μg/mL kanamycin or 50 μg/mL apramycin, as required. *E coli* DH5α was propagated on Luria‐Bertani agar or in broth with the appropriate antibiotic. When necessary, HBA was supplemented with 0.25% 2‐DOG.

### 2.2 Protein analyses

Whole cell lysates were resuspended in 2× Laemmli Buffer (Sigma) and boiled for 5 minutes. Proteins were visualized by Western immunoblotting after sodium dodecyl sulfate polyacrylamide gel electrophoresis (SDS‐PAGE) on precast polyacrylamide protein gels (NuPAGE 4%‐12% Bis‐Tris/MES, Invitrogen), as described previously.^16^ Proteins were transferred onto nitrocellulose membrane using the iBLOT (Life Technologies). *H pylori* proteins were reacted with rabbit anti‐CagA serum 17 (1:2000 dilution). Secondary alkaline phosphatase‐conjugated goat anti‐rabbit antibodies and NBT/BCIP (Nitro blue tetrazolium chloride/5‐Bromo‐4‐chloro‐3‐indolylphosphate, Sigma) were used to detect antibody‐CagA complexes.

### 2.3 Molecular biology techniques and plasmid construction

Phusion High‐Fidelity DNA polymerase (Finnzymes), DNA‐modifying enzymes (Fermentas), plasmid mini‐prep, genomic DNA extraction, and PCR/gel extraction kits (Qiagen) were used according to the manufacturer's instructions. Oligonucleotides were synthesized by Alta Bioscience, Birmingham. Where necessary, plasmids were maintained and propagated from *E coli* DH5α. DNA was sequenced by the Sanger method by the University of Birmingham functional genomics facility. Plasmids and oligonucleotides used in this study are listed in Table 1 and Table S2, respectively. To construct pCagAKO, an overlap PCR approach was used. For this, DNA fragments encoding the 3′ and 5′ ends of the CagA gene were amplified using primers CagA3′EcoRI_F with CagA3′OE_R and CagA5′OE_F with CagA5′BamHI_R, respectively. In each reaction, one of the primers contained 30 nucleotides of complementary CagA sequence so that both primers encoded a homologous region of the DNA sequence flanking *cagA* (overlap). *H pylori* B128 7.13 genomic DNA was used as the PCR template. Two PCR fragments were combined in a subsequent overlap PCR reaction with CagA3′EcoRI_F and CagA5′BamHI_R primers, generating a 916 bp fragment. This fragment was digested with EcoRI‐BamHI and ligated into pAERP3, digested with the same enzymes, to generate plasmid pCagAKO.

**Table 1:** Bacterial strains and plasmids used in this study

Strain or plasmid	Relevant genotype or sequence	Reference
Bacterial strains		
*Escherichia coli* DH5α	*fhuA2 Δ(argF‐lacZ)U169 phoA glnV44 Φ80 Δ(lacZ)M15 gyrA96 recA1 relA1 endA1 thi‐1 hsdR17*	NEB
*Helicobacter pylori* B128 7.13	*cag*PAI^+^/T4SS^+^	22
Bacterial plasmids		
pAERP2	Mutagenesis gene module containing DNA sequence to insert the 2W1S epitope into *napA* (Apra^r^)	This study
pAERP3	Mutagenesis gene module containing DNA sequence to insert the 2W1S epitope into *cagA* (Kan^r^)	This study
pCagAKO	pAERP3 derivative containing DNA sequence to delete *cagA* cloned between *Eco*RI‐*Bam*HI sites	This study

### 2.4 Gene synthesis

GeneArt (ThermoFisher Scientific) or Gene Synthesis (Biobasic) and the backbone vectors pUC57 and pMA were used where indicated for codon optimization for *H pylori* and for de novo DNA synthesis, respectively. The GenBank accession number for the protein sequence of GalK in this study is ABC98037.1.

### 2.5 Generation of isogenic mutants using counter‐selection

Approximately 500 ng of plasmid DNA was transformed into *H pylori* B128 7.13 by electroporation, as described previously.^18^ Transformants were selected on HBA plates, containing the appropriate antibiotic (positive selection). Following a 4‐ to 5‐day incubation period, 4‐6 antibiotic‐resistant colonies were patch plated onto fresh HBA plates containing the appropriate antibiotic and incubated for 1‐2 days. Patches were then passaged onto half of a fresh HBA plate, with no selection. The following day, *H pylori* growth was collected using a sterile cotton swab, suspended in 0.5 mL of BHI broth and 0.1 mL aliquots were plated onto HBA plates supplemented with 0.25% 2‐DOG (negative selection). Following a 4‐ to 5‐day incubation period, 2‐3 colonies were picked from each of the original 4‐6 antibiotic‐resistant colonies and patched onto fresh HBA plates containing 0.25% 2‐DOG. The following day, PCR was performed on each patch to confirm the presence of the desired mutation. To do this, growth was harvested from one edge of the patch and resuspended in 50‐100 µL of 10% Chelex solution (10% Chelex 100 resin [BioRad], 50 mM Tris, pH 10.5). This suspension was incubated at 56°C for 30 minutes and centrifuged at 11 000 *g* for 1 minute to pellet the Chelex beads. Supernatant (40‐90 µL) containing bacterial DNA was transferred to a new tube and 1‐2 µL was used immediately in a PCR, or stored at −20°C. The remainder of the *H pylori* growth was passaged onto a HBA plate, without selection. The following day, growth from colonies that were positive by PCR screening were harvested and stored at −80°C in 15% BHI‐glycerol medium.

### 2.6 Genome sequencing

Whole genomes were sequenced by MicrobesNG, University of Birmingham using the MiSeq Illumina platform. The Pacific Biosciences (PacBio) platform was used for de novo sequencing of *H pylori* B128 7.13 by the Centre for Genomic Research (CGR), University of Liverpool. Briefly, *H pylori* B128 7.13 genomic DNA was extracted using the Qiagen Blood DNeasy kit, according to the manufacturer's instructions. An average of 10 µg of DNA was supplied to CGR for sequencing. Supplied material was purified with 1× cleaned Ampure beads (Agencourt), and the quantity and quality were assessed using Nanodrop and Qubit assays. In addition, the fragment analyzer (using a high sensitivity genomic kit) was used to determine the average size of the DNA. The DNA was used directly without shearing. DNA was treated with Exonuclease V11 at 37**°**C for 15 minutes. The ends of the DNA were repaired as follows. Samples were incubated for 20 minutes at 37**°**C with damage repair mix supplied in the SMRTbell library kit (Pac Bio). This was followed by a 5‐minute incubation at 25**°**C with end repair mix. DNA was cleaned using 1:1 volume ratio of Ampure beads and 70% ethanol washes. DNA was ligated to adapter overnight at 25**°**C. Ligation was terminated by incubation at 65**°**C for 10 minutes followed by exonuclease treatment for 1 hour at 37**°**C. The SMRTbell library was purified with 1:1 volume ratio of Ampure beads. Size selection was performed on Sage blue pippin prep using 0.75% agarose cassette and S1 marker using a cutoff of 6 kb. The final SMRT bell was recovered as before and quantified by Qubit assay and fragment analyzer. SMRTbell library was annealed to sequencing primer at values predetermined by the Binding Calculator (Pac Bio) and a complex made with the DNA polymerase (P6/C4 chemistry). The complex was bound to Magbeads, and this was used to set up the required number of SMRT cells for the project. Sequencing was done using 360 minute movie times.

### 2.7 Bioinformatic analyses

PacBio reads were assembled de novo with Canu v1.7,^19^ which generated five contigs. Four of these were the B128.713 genome and one was the B128.7.13 plasmid. The contigs for the genome were conitguated into a single scaffolded genome sequence using Pilon.^20^ The draft genome was visualized and verified using Gap5.^21^ The draft genome was polished using MIRA v4.0.2 in mapping mode using Illumina reads from the same sample. Both the genome and plasmid were annotated using PROKKA v1.12.^22^ DNA sequences flanking the targeted sites for mutagenesis were analyzed using BLASTN and visualized in EasyFig.^23^ Global genome alignments were visualized using Mauve. All genomic sequences have been deposited to GenBank (BioProject PRJNA491697).

## 3 RESULTS

### 3.1 Genome sequence of B128 7.13

**Figure 1:**
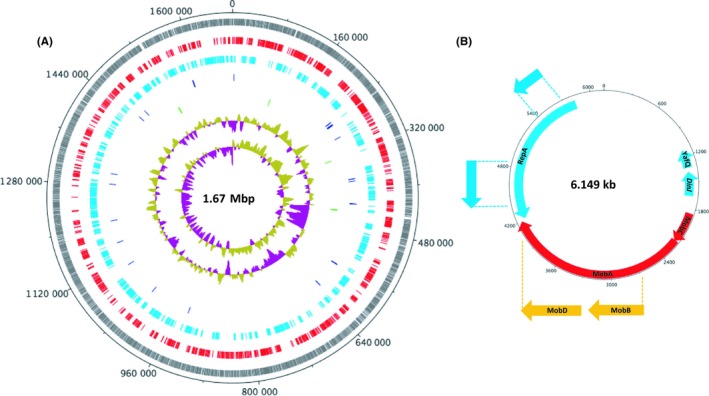
Circular representation of the *Helicobacter pylori* B128 7.13 genome. The B128 7.13 genome consists of one circular chromosome (1.67 Mbp) and one plasmid (6.149 kb). A, The chromosome is illustrated showing (from the outside to inside) the sizes in bp (circle 1); all open reading frames in grey (circle 2); the 1584 identified forward (red) and reverse (cyan) ORFs, respectively (circle 3 and 4); the tRNAs (blue) and rRNAs (green), respectively (circles 5 and 6); the plot of G + C content (circle 7) and the plot of the G + C skew (circle 8). The genome was found to have a G + C content of 38.8%. B, The plasmid has nine open reading frames homologous to the ORFs in the *H pylori* B8 plasmid. These include (clockwise) two ORFs with homology to the dinJ‐yafQ toxin‐antitoxin (TA) system (cyan), a cluster of conjugation mobilization proteins mobC (red), mobA (red), mobB (orange), and mobD (orange), a replication initiation protein A (repA) (cyan), and two uncharacterized ORFs (blue)

*Helicobacter pylori* strain B128 was originally isolated from a human patient suffering from gastric ulcers.^14^ B128 was subsequently used to challenge a Mongolian gerbil, which resulted in the recovery of an in vivo adapted strain that was able to induce adenocarcinoma in gerbils and was designated strain, B128 7.13.^24^ Furthermore, in an independent experiment, B128 was used to infect Mongolian gerbils for a total of three times, and a resulting strain, named B8, was recovered.^25^ The genome sequence of B8 has been assembled and this strain is routinely used as a reference genome for investigators interested in the biology of B128 7.13.^26^ Although the draft genome of B128 7.13 has been reported using Illumina sequencing technology,^26^, ^27^ the closed genome of this strain has not been reported. Given that we sought to confirm the mutations present in our mutagenized B128 7.13 strains on a genome‐wide scale and given the importance of the B128 7.13 strain to the wider research community, we used both the PacBio and Illumina sequencing platforms to determine the closed genome sequence of B128 7.13. The genome consists of a circular chromosome of 1, 675, 441 bp, as well as a 6,149 bp plasmid, which was not reported in the draft genomes of B128 7.13 (Figure 1).

Except for a 117 bp duplication in a noncoding region, the B128 7.13 plasmid is 100% identical to the HPB8p plasmid from *H pylori* B8.^25^ This high level of identity is expected given that B128 7.13 and B8 are both derived from the same progenitor strain, B128. From herein, the plasmid in strain B128 7.13 is named pHP7.13. Our initial annotation of pHP7.13 identified only four coding sequences (CDS), as opposed to the annotation of B8, for which nine CDS were identified.^25^ This is most likely due to differences in the method of annotation as PROKKA may be unable to detect the additional five CDS due to their small sizes and localization as nested CDS. Thus, we manually annotated pHP7.13 to highlight the nine CDS that have been functionally identified,^25^ including a cluster of conjugation mobilization proteins (*mobA‐D*); a replication initiation protein A (*repA*); two CDS with homology to the *dinJ‐yafQ* toxin‐antitoxin (TA) system, described in *E coli* 28; and two uncharacterized CDS that are specific to plasmids, pHPB8 and pHP7.13, found in *H pylori* B8 and B128 7.13, respectively (Figure 1).

In the B128 7.13 chromosome, we identified 1584 CDS, which included 622 hypothetical genes and 36 tRNA, 4 rRNA, and 1 tmRNA genes. Consistent with previous draft genomes of B128 7.13, the genome has a G + C content of 38.8%. Due to the long sequence reads generated by the PacBio sequencing technology, we were able, for the first time to, visualize global genome alignments of our B128 7.13 genome with the strain B8 reported in Farnbacher et al.^25^ Overall, the genomes aligned almost perfectly (Figure S1), the largest region of difference being an insertion of a 1430 bp at position 1414705 in B128 7.13. This insertion encodes a duplication of a *H pylori‐*specific DUF874 family protein of unknown function but which is thought to encode a DNA‐binding protein that plays a role in stress tolerance and survival in vivo.^29^ For the purpose of this study, we have not provided a full annotation of the B128 7.13 genome, but have rather used it as the reference genome for our mutagenized strains generated by the methods described below.

### 3.2 Generation of a mutagenesis gene module for targeted mutagenesis

**Figure 2:**
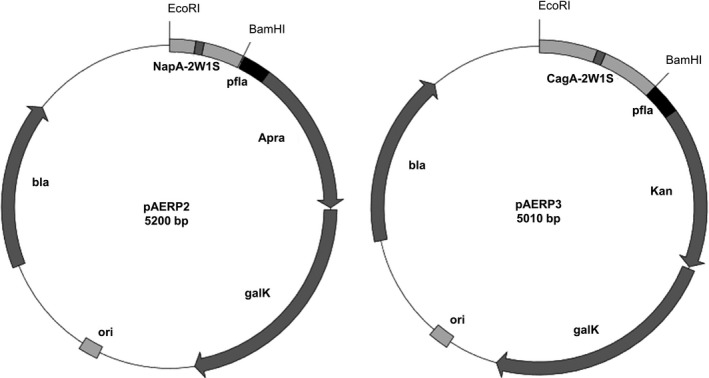
Mutagenesis plasmids pAERP2 and pAERP3 used in this study. Both plasmids have the *Escherichia coli galK* gene and either the genes encoding apramycin (Apra) or kanamycin (Kan) resistance under the control of the *H pylori flaA* flagellin promoter (pfla). Both plasmids contain the target mutations upstream of the pfla promoter labeled NapA‐2W1S (pAERP2) or CagA‐2W1S (pAERP3). In pAERP2, NapA‐2W1S contains a 330 bp region of homology to the DNA sequence flanking the start codon of the neutrophil‐activating protein (NapA) (light grey) which is interrupted by a 42 bp sequence encoding the 2W1S epitope (dark grey) inserted at amino acid position 2 in NapA. In pAERP3, CagA‐2W1S contains a 561 bp region of homology to the DNA sequence flanking the start codon of the CagA protein (light grey), which is interrupted by a 42 bp sequence encoding the 2W1S epitope (dark grey) inserted at amino acid position 3 in CagA. Restriction sites for enzymes EcoRI and BamHI are shown. The pAERP2 and pAERP3 plasmids were used to generate an in‐frame insertion of the 2W1S epitope into the coding regions of *napA* and *cagA*, respectively

To generate the mutagenesis plasmids pAERP2 and pAERP3 (shown in Figure 2), DNA was synthesized de novo and codon optimized for expression in *H pylori* by GeneArt Synthesis (Thermo Fisher Scientific) and Gene Synthesis (BioBasic), respectively. Both plasmids contain the mutagenesis gene module comprising the 144‐bp *H pylori flaA* promoter^13^ upstream of either the apramycin resistance cassette (pAERP2) or the kanamycin resistance cassette (pAERP3) and the *galK* gene from *E coli* 30 (Figure 2). DNA fragments containing the target mutation of interest were flanked between the indicated restriction sites and were located immediately upstream of the *flaA* promoter. Plasmids pAERP2 and pAERP3 contain DNA sequences, named NapA‐2W1S and CagA‐2W1S (Figure 2), comprising 331 and 561 bp regions of homology flanking the start ATG codons of *napA* (neutrophil‐activating protein)^31^ and *cagA* (cytotoxin‐associated gene A)*,* 32 respectively. In addition to these regions of homology, NapA‐2W1S and CagA‐2W1S DNA sequences contained 42 bp insertions encoding a 14‐amino acid epitope (2W1S) with the sequence EAWGALANWAVDSA, which is recognized by a subpopulation of T cells in C57BL/6 mice.^33^, ^34^ However, for the purpose of this study, the 2W1S epitope is simply used as a marker for the modification of the B128 7.13 chromosome. After recombination, the 2W1S epitope was inserted after amino acid positions 2 and 3 of NapA and CagA, respectively. Annotated sequences of the mutagenesis gene modules in pAERP2 and pAERP3 are shown in Figure S2.

In order to show the feasibility of using this method to mediate markerless, chromosomal gene deletions, we constructed the plasmid pCagAKO from pAERP3, as described in the Materials and Methods. The annotated sequence of pCagAKO is shown in Figure S2.

### 3.3 Generation of isogenic mutants using a one‐step transformation and counter‐selection method

**Figure 3:**
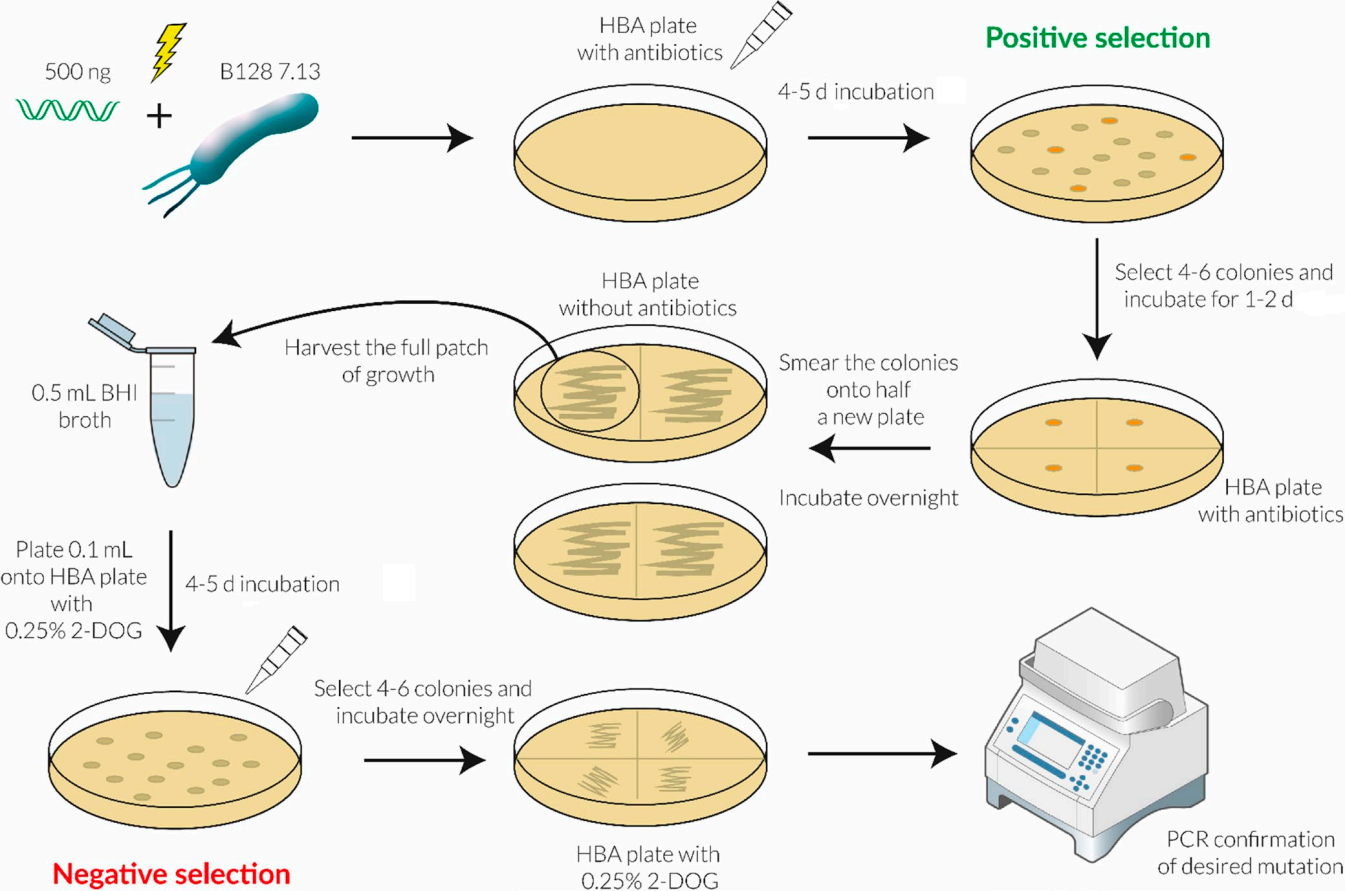
Schematic illustration of the mutagenesis strategy used in this study. Briefly, approximately 500 ng of plasmid DNA was transformed into *H pylori* electrocompetent cells. Transformants were selected on HBA plates containing the appropriate antibiotic, and 4‐6 colonies were patch plated onto fresh HBA plate containing antibiotic. These patches were then spread onto half a HBA plate with no selection, and after a 1‐day incubation period, growth was resuspended in 0.5 mL of BHI broth. 0.1 mL aliquots were then plated onto HBA containing 0.25% 2‐DOG. After 4‐ to 5‐day incubation, 4‐6 colonies from each original antibiotic‐resistant colony were patch plated onto HBA containing 0.25% 2‐DOG and each patch was tested by PCR for the desired mutation

**Figure 4:**
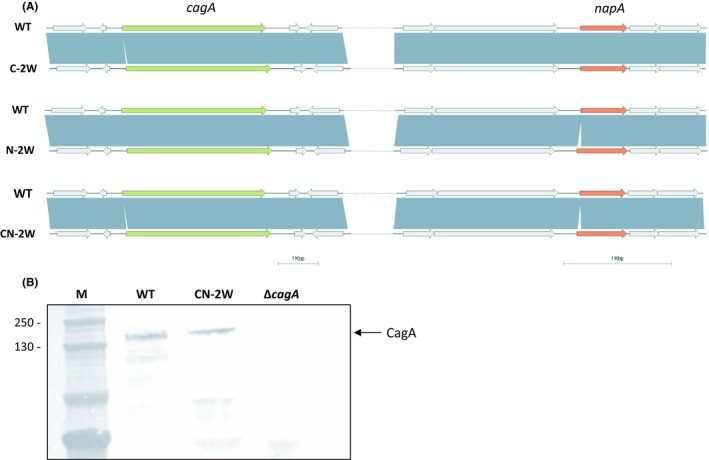
Comparison of mutant strains with the B128 7.13 parental strain, using nucleotide alignments and Western immunoblotting. A, Nucleotide alignments and open reading frame (ORF) representation of the parental strain B128 7.13 (WT) and mutant strains that carry an N‐terminal insertion of the 2W1S epitope in *cagA* (C‐2W), *napA* (N‐2W) or both *cagA* and *napA* (CN‐2W) are illustrated using Easyfig.^29^ For simplicity, ORFs flanking *cagA* (green arrow) and *napA* (orange arrow) are unlabeled and shown as light grey arrows. Dark grey bands between the indicated strains denote regions of identity. White gaps mark the insertion of the sequence encoding the 2W1S epitope in the N‐terminal region of *cagA* (C‐2W), the N‐terminal region of *napA* (N‐2W) and in both the N‐terminal regions of *cagA* and *napA* (CN‐2W), as a result of the targeted mutagenesis strategy. B, Western immunoblotting analyses of CagA production in cell lysates of the B128 7.13 parental strain (WT), the strain that contains N‐terminal insertion of 2W1S in the CN‐2W strain and the strain where CagA is deleted (Δ*cagA*). CagA localization was detected using anti‐CagA antibody and is indicated by an arrow at the molecular weight of approximately 150 kDa (M—protein markers, kDa). As expected, CagA is present in the parental B128 7.13 strain and the CN‐2W strain, yet is absent from the Δ*cagA* strain generated in this study. These results confirm that the mutagenesis strategy has inserted an in‐frame, 2W1S epitope at the N‐terminus of CagA in the C‐2W and CN‐2W strain, without disrupting the production of full‐length CagA protein and has successfully deleted *cagA* in the Δ*cagA* deletion strain

To investigate whether the mutagenesis gene module containing *galK* can be used as a counter‐selection strategy in *H pylori*, we transformed plasmids pAERP2 and pAERP3 into the *H pylori* strain B128 7.13. A schematic illustration of this method is shown in Figure 3. pAERP2 and pAERP3 were designed to insert the 2W1S epitope into the N‐terminal region of NapA and CagA, respectively. Plasmids pAERP2 and pAERP3 do not contain an origin of replication compatible with *Helicobacter* spp., and so apramycin or kanamycin resistance can only be selected for through homologous recombination in which the entire plasmid is integrated onto the chromosome at the targeted mutagenesis site. The 2W1S epitope in plasmids pAERP2 and pAERP3 was flanked by regions of homology (between 0.3 and 0.6 kb) to the *napA* and *cagA* genes, respectively, generating *napA‐2W1S* and *cagA‐2W1S* alleles. Thus, this region of homology directed single crossover events at the native *napA* and *cagA* loci, resulting in a chromosomal duplication whereby the plasmid sequence encoding antibiotic resistance and the *galK* gene interrupt the native loci from the mutant alleles, as described previously.^7^ Isolated colonies were kanamycin or apramycin resistant and sensitive to 2‐DOG. Antibiotic‐resistant isolates were grown in the presence of 2‐DOG to select for excision of the plasmid sequence and resulted in two outcomes: resolution to either the native allele or the mutant allele. In either scenario, isolates were kanamycin or apramycin sensitive and 2‐DOG resistant. Multiple colonies were then verified for the veracity of mutations by PCR and, where indicated, by whole genome sequencing (Figure 4A). For each strain, the number of antibiotic‐sensitive and 2‐DOG‐resistant colonies tested for successful recombination to the mutant allele is shown in Table S5. On average, 59% of the screened colonies were positive for the desired mutation. Positive clones by PCR were then submitted to MicrobesNG for whole genome sequencing, and the resulting sequences were compared to the B128 7.13 parental strain. From herein, strains in which the 2W1S epitope was successfully inserted into CagA and NapA will be called C‐2W and N‐2W, respectively.

To test the compatibility of this method for multiple genomic modifications within the same strain, we used this system to generate a strain whereby both CagA and NapA are marked with the 2W1S epitope. To do this, N‐2W was transformed with pAERP3 in order to insert into *cagA* the sequence encoding the 2W1S epitope. Kanamycin‐sensitive and 2‐DOG‐resistant colonies were isolated using the counter‐selection method described above. The veracity of the double mutant strain, named CN‐2W, was confirmed by whole genome sequencing (Figure 4A). Furthermore, production of CagA in whole cell lysates prepared from C‐2W and CN‐2W was confirmed by Western immunoblotting, using antibodies raised against CagA (Figure 4B). In order to ensure that the mutagenesis method did not cause significant secondary site mutations, VarScan was used to identify single nucleotide polymorphisms (SNPs) and small insertions or deletions (indels) between the B128 7.13 parental strain and C‐2W, N‐2W, and CN‐2W. Analyses were performed using a stringent 90% cutoff value, which required at least 90% of sequence reads to contain the SNP. A total of eight SNPs were identified common to all three sequenced strains, of which six were found in coding regions. Of these six SNPs: four caused missense mutations in the genes *gpsA,* HP713_00241, and HP713_01483; one caused a frame shift in *cysP_2*; and one caused a premature stop codon in *fliF* (Table S2). However, these mutations were not found in all B128 7.13 isogenic mutants made in our laboratory using this method (data not shown), suggesting that these mutations are not a result of this mutagenesis protocol. SNPs were also found that were unique to strains C‐2W, N‐2W, and CN‐2W. A complete list of SNPs and their locations can be found in Table S2. Importantly, no significant genomic inversions or indels were observed as a result of this mutagenesis method. Moreover, the flanking and coding sequences of *cagA* and *napA* remained identical to those of the parental strain, showing that secondary site mutations are not an issue with this mutagenesis method (Figure 4A).

In order to use this method to delete *cagA* in B128 7.13, pCagAKO was transformed into *H pylori* B128 7.13 following the mutagenesis protocol above. To confirm the loss of *cagA,* primers that annealed to the central portion of *cagA* were used in PCRs to screen candidate isolates that were kanamycin sensitive and 2‐DOG resistant (data not shown). An isolate that was confirmed by PCR for the absence of *cagA* was also tested using Western immunoblotting to ensure the absence of CagA in whole cell lysates (Figure 4B).

## 4 DISCUSSION

Here, we reveal the closed genome sequence of *H pylori* strain B128 7.13 and a method for generating genomic markerless and scarless isogenic mutants. *H pylori* B128 7.13 is an important strain for the study of *H pylori* physiology, both in vitro and in vivo.^14^, ^26^ Consistent with previous reports of *H pylori* genomes, including the draft genomes of B128 7.13 that were recently published,^14^, ^26^, ^27^ the B128 7.13 genome size is relatively small (1.67 Mbp) in comparison to other gastrointestinal pathogens, such as *E coli* 35 and *S.* Typhimurium,^36^ where larger genomes are typically observed (5‐6 Mbp). The comparatively small genome size of *H pylori* is likely due to extensive adaptation to the human stomach over thousands of years, and we refer the reader to the extensive literature discussing this topic.^37^, ^38^ The G + C content of the B128 7.13 genome was found to be 38.8% with 1584 CDS. The annotation described by Noto et al showed a lower number (1485) of CDS (Table S1), which could be due to differences in the programs used for annotation. Alternatively, this difference could be attributed to the long‐read sequencing technology used here vs the limitations of the short‐read technology available previously*.* For example, the early termination of genes due to short reads may lead to an increase in the numbers of pseudogenes identified and therefore a reduction in true CDS. A notable finding of the current work was a 1430 bp insertion present in the genome of B128 7.13, which is absent in strain B8. This insertion encodes a duplication of an *H pylori*‐specific DUF874 family protein, which has been implicated in survival of *H pylori* in a mouse model^29^ and thus could have arisen during adaptation of B128 to the gerbil.

The mutagenesis approach taken in this study involves one cloning step and a single transformation, followed by positive and negative selection to isolate markerless and scarless isogenic mutants of B128 7.13. Although the counter‐selectable marker *sacB,* which confers sensitivity to sucrose, has been used by multiple groups to generate scarless mutants in *H pylori*,^2^, ^10^, ^39^ there have been reports of *sacB* inactivation and spontaneous resistance to sucrose in *H pylori* and other bacteria.^11^, ^40^, ^41^ This has prompted the use of alternative markers, such as *galK,* which does not seem to be as prone to inactivation as *sacB*.^42^ Nevertheless, this has not been systematically studied in *H pylori* and therefore warrants further investigation. Given that transformation efficiency can be influenced by the antibiotic resistance cassette used for selection in *H pylori*, we synthesized plasmids pAERP2 and pAERP3 to encode the kanamycin and apramycin resistance cassettes, respectively. However, no significant differences were observed between transformation efficiencies of B128 7.13 with either pAERP2 or pAERP3 (data not shown). The mutagenesis gene modules in both pAERP2 and pAERP3 comprise the strong, constitutive *flaA* promoter, which mediates expression of the respective antibiotic resistance cassette and the *galK* gene (Figure 2). The EcoRI and BamHI restriction sites upstream of the mutagenesis gene module enable cloning of DNA sequences homologous to the target loci of interest. In our hands, 0.3‐0.6 kb of DNA sequence homologous to the target loci (Figure 2) was sufficient to mediate homologous recombination and subsequent integration of the entire plasmid at the chromosomal locus. However, increasing the length of DNA sequences (up to 1 kb) has been shown to increase the frequency of homologous recombination in bacteria.^43^ Using this method, we were able to isolate mutant strains that contain in‐frame insertions in CagA (C‐2W), NapA (N‐2W) and both CagA and NapA (C/N‐2W). Furthermore, using Western immunoblotting, we confirmed that C‐2W and C/N‐2W were still able to produce full‐length CagA (Figure 4). Additionally, in order to create an isogenic mutant of B128 7.13 lacking *cagA*, we modified pAERP3 to generate the plasmid pCagAKO (Figure 4). Thus, we present here a versatile method that can be used to generate multiple genomic modifications in B128 7.13. To confirm the veracity of mutagenized strains, whole genome sequences of C‐2W, N‐2W, and C/N‐2W were analyzed bioinformatically to ensure that there were no significant second site mutations or large genomic rearrangements. Although no large genomic rearrangements or inversions were evident in our analyses, VarScan did highlight a number of SNPs that were either unique to each derived strain or were common between the strains. The eight SNPs that were found to be common between C‐2W, N‐2W, and C/N‐2W strains may reflect mutations that are more frequently selected for during growth under standard laboratory conditions. For example, the SNP present in *fliF*, which encodes a component of the flagellar machinery, may be an adaptation to growth in vitro. All SNPs are listed in Table S3; most importantly, these SNPs do not appear in all the strains that our laboratory has generated using this method (data not shown). This method is advantageous in comparison with alternative mutagenesis approaches given that only one cloning step and one transformation step are required to generate scarless mutations. However, the efficiency of recombination to the mutant allele using this method is on average 59%, which is lower than that of previously reported methods.^11^, ^12^ For comparison, we have listed the reported efficiencies and advantages and disadvantages of the available mutagenesis methods in *H pylori* using counter‐selectable markers (Table S4). The main limitation of this method is that we were unable to generate mutants in the mouse‐colonizing *H pylori* SS1 and PMSS1 strains,^44^ and although kanamycin‐sensitive and 2‐DOG‐resistant colonies could be isolated, they did not contain the desired mutations (data not shown). It is generally known that SS1 and PMSS1 are not as easily amenable to genetic manipulation as other *H pylori* strains.^5^ Nevertheless, given that antibiotic‐resistant and 2‐DOG‐resistant colonies were isolated, it is likely that, with further optimization, this method may be used to generate mutants in this and other less genetically tractable strains*.*

In summary, we have established a method to generate isogenic mutants of *H pylori* strain B128 7.13. We have shown that when using this mutagenesis method, the mutants have no second site mutations within the flanking regions of the target mutation, have limited SNPs and/or indels, do not contain an antibiotic marker, and are amenable to successive rounds of genomic modifications. Furthermore, we report the closed genome of B128 7.13, which provides a useful resource for researchers interested in the biology of B128 7.13, which is one of the reference strains commonly used in mouse infection studies.

## DISCLOSURES OF INTERESTS

The authors have no disclosures or other conflicts of interest to report.
