# Supplementary material for: Complete genome sequence of Helicobacter pylori B128 7.13 and a single‐step method for the generation of unmarked mutations
Source: Helicobacter. 2019 May 7;24(4):e12587. doi: 10.1111/hel.12587 (PMC6618122; doi:10.1111/hel.12587)
Supplement: Supplementary file 1 [file HEL-24-na-s001.pdf]

(A)

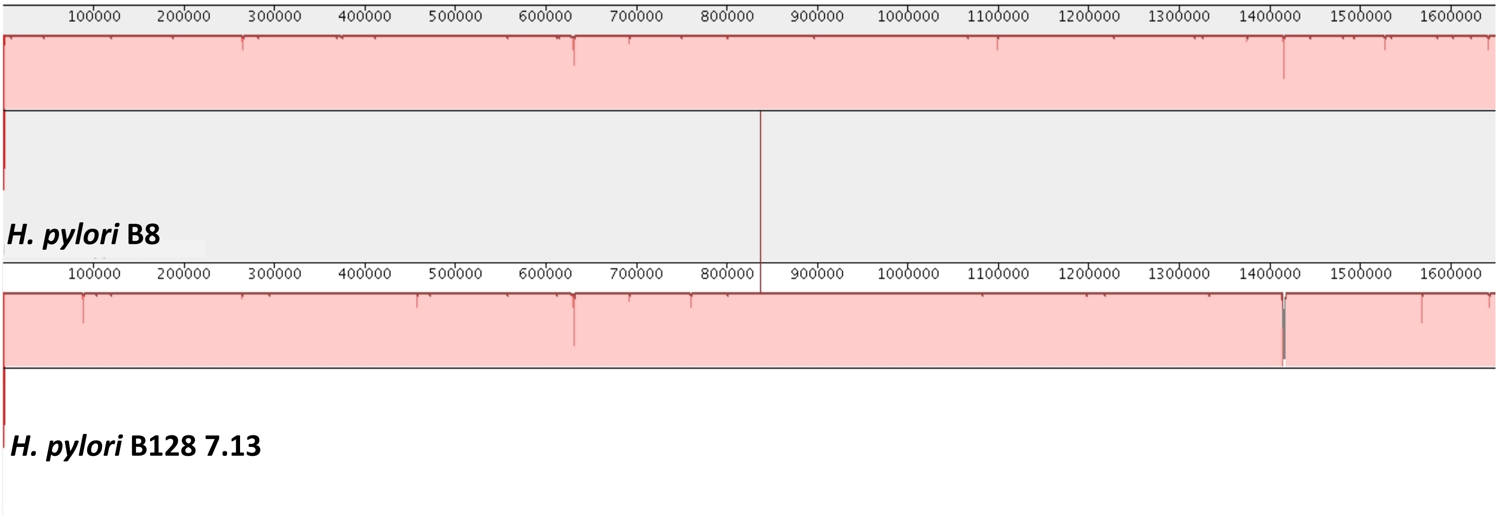

(B)

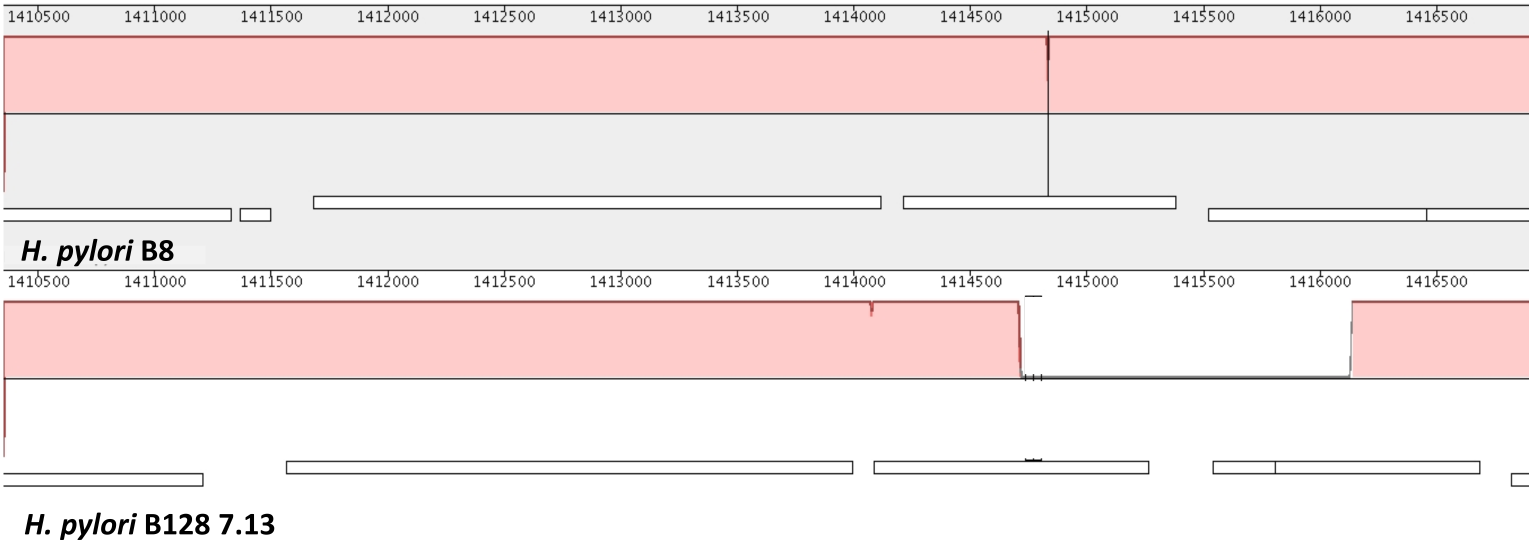

Supplementary Figure 1. Alignments of the *H. pylori* B8 and B128 7.13 genomes. (A) Global genome alignment of *H. pylori* B8 (top) and B128 7.13 (bottom), visualised using Mauve. Red bands between the indicated genomes denote regions of identity. Red troughs indicate regions of difference between the two genomes, with the most significant trough representing an inversion in the B128 7.13 genome at position 1414705. This inversion is shown in more detail in panel B. (B) Zoomed in alignment of the insertion present in B128 7.13, represented as the white gap compared to B8. The insertion encodes a duplication of the DUF874 gene, a *H. pylori*-specific protein thought to be involved in survival in a mouse model of infection (27).
