# Supplementary material for: Complete genome sequence of Helicobacter pylori B128 7.13 and a single‐step method for the generation of unmarked mutations
Source: Helicobacter. 2019 May 7;24(4):e12587. doi: 10.1111/hel.12587 (PMC6618122; doi:10.1111/hel.12587)
Supplement: Supplementary file 2 [file HEL-24-na-s002.pdf]

Figure S2. Annotated sequences of the mutagenesis gene module in plasmids pAERP2 (A) and pAERP3 (B) and pCagAKO (C). **A:** EcoRI sequence is shown in bold black font. DNA sequence homologous to *napA* is shown in blue font and the ATG start codon of NapA is shown in uppercase red font. The sequence encoding the 2W1S epitope is shown in uppercase black font. The BamHI restriction site is shown in bold purple. The flaA promoter sequence is shown in red. The sequence encoding the apramycin resistance cassette is shown in upper case black font. The sequence encoding the *E. coli galK* gene is shown in upper case green font. **B:** EcoRI sequence is shown in bold black font. DNA sequence homologous to *cagA* is shown in blue font and the ATG start codon of CagA is shown in uppercase red font. The sequence encoding the 2W1S epitope is shown in uppercase black font. The BamHI restriction site is shown in bold purple. The flaA promoter sequence is shown in red. The sequence encoding the kanamycin resistance cassette is shown in upper case black font. The sequence encoding the *E. coli galK* gene is shown in upper case green font. **C:** EcoRI sequence is shown in bold black font. DNA sequence homologous to the 3'and 5' flanking regions of *cagA* are shown in blue and orange, respectively. The stop codon (TAA) and the start codon (ATG) of *cagA,* within these flanking regions, are shown in uppercase. The BamHI restriction site is shown in bold purple. The flaA promoter sequence is shown in red. The sequence encoding the apramycin resistance cassette is shown in upper case black font. The sequence encoding the *E. coli galK* gene is shown in upper case green font.

A

>pAERP2
gaattcgtttatcaatatttgacaaaaataagatcaaaacaatttttcattaactcttttgg
tgtaggatagcgatcaaggttttatgaaaataaaagcctaaaacaattttaaaaaaaggac
ttttgATGGAGGCGTGGGAGCGCTAGCGAACTGGGCGGTAGACTCAGCGaaaacatttgaa
attttaaaacatttgcaagcggatgcgatcgtgttatttatgaaagtgcataacttccattg
gaatgtgaaaggcaccgatttttcaatgtgcataaagccactgaagaaatttatgaagaat
ttgcggacatgtttgatgatctcgctgaaaggatcgctcaattagggcaccaccccttagtc
acttta**ggatcc**ggtataagcccattttcatgctcctaattttgcttttaaaataaagccct
ttaaaatttcaaactttaaccgataatagtttcaaccaaaagcaaggatgcctttgggtttt
ttataaggatcgtgactaactaggaggaataaATGCAATACGAATGGCGAAAAGCCGAGCTC
ATCGGTCAGCTTCTCAACCTTGGGGTTACCCCCGGCGGTGTGCTGCTGGTCCACAGCTCCTT
CCGTAGCGTCCGGCCCCTCGAAGATGGGCCACTTGGACTGATCGAGGCCCTGCGTGCTGCGC
TGGGTCCGGGAGGGACGCTCGTCATGCCCTCGTGGTCAGGTCTGGACGACGAGCCGTTCGAT
CCTGCCACGTCGCCCGTTACACCGGACCTTGGAGTTGTCTCTGACACATTCTGGCGCCTGCC
AAATGTAAAGCGCAGCGCCCATCCATTTGCCTTTGCGGCAGCGGGGCCACAGGCAGAGCAGA
TCATCTCTGATCCATTGCCCCTGCCACCTCACTCGCCTGCAAGCCCGGTCGCCCGTGTCCAT
GAACTCGATGGGCAGGTACTTCTCCTCGGCGTGGACACGATGCCAACACGACGCTGCATCT
TGCCGAGTTGATGGCAAAGGTTCCCTATGGGGTGCCGAGACACTGCACCATTCTTCAGGATG
GCAAGTTGGTACGCGTCGATTATCTCGAGAATGACCACTGCTGTGAGCGCTTTGCCTTGGCG
GACAGGTGGCTCAAGGAGAAGAGCCTTCAGAAGGAAGGTCCAGTCGGTCATGCCTTTGCTCG
GTTGATCCGCTCCCGCGACATTGTGGCGACAGCCCTGGGTCAACTGGGCCGAGATCCGTTGA
TCTTCCTGCATCCGCCAGAGGCGGGATGCGAAGAATGCGATGCCGCTCGCCAGTCGATTGGC
TGAtacctggagggaataATGAGTCTGAAAGAAAAAACACAATCTCTGTTTGCCAACGCATT
TGGCTACCCTGCCACTCACACCATTCAGGCGCCTGGCCGCGTGAATTTGATTGGTGAACACA
CCGACTACAACGACGGTTTCGTTCTGCCCTGCGCGATTGATTATCAAACCGTGATCAGTTGT
GCACCACGCGATGACCGTAAAGTTCGCGTGATGGCAGCCGATTATGAAAATCAGCTCGACGA
GTTTTCCCTCGATGCGCCCATTGTCGCACATGAAAACTATCAATGGGCTAACTACGTTCGTG
GCGTGGTGAAACATCTGCAACTGCGTAACAACAGCTTCGGCGGCGTGGACATGGTGATCAGC
GGCAATGTGCCGCAGGGTGCCGGGTTAAGTTCTTCCGCTTCACTGGAAGTCGCGGTCGGAAC
CGTATTGCAGCAGCTTTATCATCTGCCGCTGGACGGCGCACAAATCGCGCTTAACGGTCAGG

AAGCAGAAAACCAGTTTGTAGGCTGTAACTGCGGGATCATGGATCAGCTAATTTCCGCGCTC
GGCAAGAAAGATCATGCCTTGCTGATCGATTGCCGCTCACTGGGGACCAAAGCAGTTTCCAT
GCCCAAAGGTGTGGCTGTCGTCATCATCAACAGTAACTTCAAACGTACCCTGGTTGGCAGCG
AATACAACACCCGTCGTGAACAGTGCGAAACCGGTGCGCGTTTCTTCCAGCAGCCAGCCCTG
CGTGATGTCACCATTGAAGAGTTCAACGCTGTTGCGCATGAACTGGACCCGATCGTGGCAAA
ACGCGTGCGTCATATACTGACTGAAAACGCCCGCACCGTTGAAGCTGCCAGCGCGCTGGAGC
AAGGCGACCTGAAACGTATGGGCGAGTTGATGGCGGAGTCTCATGCCTCTATGCGCGATGAT
TTCGAAATCACCGTGCCGCAAATTGACACTCTGGTAGAAATCGTCAAAGCTGTGATTGGCGA
CAAAGGTGGCGTACGCATGACCGGCGGCGGATTTGGCGGCTGTATCGTCGCGCTGATCCCGG
AAGAGCTGGTGCCTGCCGTACAGCAAGCTGTCGCTGAACAATATGAAGCAAAAACAGGTATT
AAAGAGACTTTTTACGTTTGTAAACCATCACAAGGAGCAGGACAGTGCTGAacgaaactc

B

>pAERP3
gaattcaatccaaaccataaacgctaccctttgtaatccttgataatttataatttttgcta
taataaaaccctaactaaaatttctccttttattttagttagaccccttgaattagaattat
agtagacttgttataccttgttctaaatattgtggtatattaacaatgtttaaagacatgaa
ttgactgctcaagtgtgtagcgatttttagcagtctttgacaccaataagataccgataggt
atgaaactaggtatagtaaggagaaacaATGactGAAGCTTGGGGCGCTTTGGCTAATTGGG
CTGTGGATAGCGCTaacgaaaccattaaccaacaaccacaaaccgaagcggcttttaacccg
cagcaatttatcaataatcttcaagtggcttttattaaagttgataacgctgtcgcttcatt
tgatcctgatcaaaaaccaatcgttgataagaatgatagggataacaggcaagcttttgaga
aaatctcgcagctaagggagaaattcgctaataaagcgatcaaaaatcctaccaaaaagaat
cagtatttttcaaactttatcagtaagagcagtgatttaatcaacaaagac**ggatcc**ggtat
aagcccattttcatgctcctaattttgcttttaaaataaagccctttaaaatttcaaacttt
aaccgataatagtttcaaccaaaagcaaggatgcctttgggtttttataaggatcgtgact
aactaggaggaataaATGGCTAAAATGAGAATATCACCGGAATTGAAAAAACTGATCGAAAA
ATACCGCTGCGTAAAAGATACGGAAGGAATGTCTCCTGCTAAGGTATATAAGCTGGTGGGAG
AAAATGAAAACCTATATTTAAAAATGACGGACAGCCGGTATAAAGGGACCACCTATGATGTG
GAACGGGAAAAGGACATGATGCTATGGCTGGAAGGAAAGCTGCCTGTTCCAAAGGTCCTGCA
CTTTGAACGGCATGATGGCTGGAGCAATCTGCTCATGAGTGAGGCCGATGGCGTCCTTTGCT
CGGAAGAGTATGAAGATGAACAAAGCCCTGAAAAGATTATCGAGCTGTATGCGGAGTGCATC
AGGCTCTTTCACTCCATCGACATATCGGATTGTCCCTATACGAATAGCTTAGACAGCCGCTT
AGCCGAATTGGATTACTTACTGAATAACGATCTGGCCGATGTGGATTGCGAAAACTGGGAAG
AAGACACTCCATTTAAAGATCCGCGCGAGCTGTATGATTTTTTAAAGACGGAAAAGCCCGAA
GAGGAACTTGTCTTTTCCCACGGCGACCTGGGGGACAGCAACATCTTTGTGAAAGATGGCAA
AGTAAGTGGCTTTATTGATCTTGGGAGAAGCGGCAGGGCGGACAAGTGGTATGACATTGCCT
TCTGCGTCCGGTCGATCAGGGAGGATATCGGGGAAGAACAGTATGTCGAGCTATTTTTTGAC
TTACTGGGGATCAAGCCTGATTGGGAGAAAATAAAATACTATATTTTACTGGATGAATTGTT
TTAGtacctggagggaataATGAGTCTGAAAGAAAAAACACAATCTCTGTTTGCCAACGCAT
TTGGCTACCCTGCCACTCACACCATTCAGGCGCCTGGCCGCGTGAATTTGATTGGTGAACAC
ACCGACTACAACGACGGTTTCGTTCTGCCCTGCGCGATTGATTATCAAACCGTGATCAGTTG
TGCACCACGCGATGACCGTAAAGTTCGCGTGATGGCAGCCGATTATGAAAATCAGCTCGACG
AGTTTTCCCTCGATGCGCCCATTGTCGCACATGAAAACTATCAATGGGCTAACTACGTTCGT
GGCGTGGTGAAACATCTGCAACTGCGTAACAACAGCTTCGGCGGCGTGGACATGGTGATCAG
CGGCAATGTGCCGCAGGGTGCCGGGTTAAGTTCTTCCGCTTCACTGGAAGTCGCGGTCGGAA
CCGTATTGCAGCAGCTTTATCATCTGCCGCTGGACGGCGCACAAATCGCGCTTAACGGTCAG

GAAGCAGAAAACCAGTTTGTAGGCTGTAACTGCGGGATCATGGATCAGCTAATTTCCGCGCT
CGGCAAGAAAGATCATGCCTTGCTGATCGATTGCCGCTCACTGGGGACCAAAGCAGTTTCCA
TGCCCAAAGGTGTGGCTGTCGTCATCATCAACAGTAACTTCAAACGTACCCTGGTTGGCAGC
GAATACAACACCCGTCGTGAACAGTGCGAAACCGGTGCGCGTTTCTTCCAGCAGCCAGCCCT
GCGTGATGTCACCATTGAAGAGTTCAACGCTGTTGCGCATGAACTGGACCCGATCGTGGCAA
AACGCGTGCGTCATATACTGACTGAAAACGCCCGCACCGTTGAAGCTGCCAGCGCGCTGGAG
CAAGGCGACCTGAAACGTATGGGCGAGTTGATGGCGGAGTCTCATGCCTCTATGCGCGATGA
TTTCGAAATCACCGTGCCGCAAATTGACACTCTGGTAGAAATCGTCAAAGCTGTGATTGGCG
ACAAAGGTGGCGTACGCATGACCGGCGGCGGATTTGGCGGCTGTATCGTCGCGCTGATCCCG
GAAGAGCTGGTGCCTGCCGTACAGCAAGCTGTCGCTGAACAATATGAAGCAAAAACAGGTAT
TAAAGAGACTTTTTACGTTTGTAAACCATCACAAGGAGCAGGACAGTGCTGAacgaaactc

C
>pCagAKO
gaattcctTAAaggattaaggaatatcaaaaacgcaaaaaccaccccttgctaaaaacaagg
ggtttttaatactccttagcagaaatcccaatcgtctttagtgtttgggatgaatgctacca
attcatggtatcatatccccatacattcgtatctagcgcaggaagtgcacaaagttacgcct
ttggagatatgatgtgtgagacctgtagggaatgcgttggagctcaaactctgtaaaatccc
tatgatagggacacagagtgagaaccaaattctccctacgggcaacatcagcctaggaagcc
caatcgtctttagcggttgggcgcttcaccttaaaatatcccgacagacactaacgcatatg
gctaccaaagtctttgggcgtgtggtgcgattctttctctataacggcgtctttaacacaag
caacacgcaaagcgtcaaaataagtcccaacgctagcgcaaccgccgagtaaagacgctcca
ataaacatgctatttctaatggttttcattttatatccttttgttttaaatttttaataac
tcaaatactttaatcatgcgtttatgatagttaagatttattattaaacaaaaagtaaatga
aactcaaaacaatcacaataacgccctaaatccaaaccataaacgctaccctttgtaatcct
tgataatttataatttttgctataataaaccctaactaaaatttctccttttattttagtt
agaccccttgaattagaattatagtagacttgttataccttgttctaaatattgtggtatat
taacaatgtttaaagacatgaattgactgctcaagtgtgtagcgatttttagcagtctttga
caccaataagataccgataggtatgaaactaggtatagtaaggagaaacaATG**ggatcc**ggt
ataagcccattttcatgctcctaattttgcttttaaaataaagccctttaaaatttcaaact
ttaaccgataatagtttcaaccaaaagcaaggatgcctttgggtttttataaggatcgtga
ctaactaggaggaataaATGGCTAAAATGAGAATATCACCGGAATTGAAAAAACTGATCGAA
AAATACCGCTGCGTAAAAGATACGGAAGGAATGTCTCCTGCTAAGGTATATAAGCTGGTGGG
AGAAAATGAAAACCTATATTTAAAAATGACGGACAGCCGGTATAAAGGGACCACCTATGATG
TGGAACGGGAAAAGGACATGATGCTATGGCTGGAAGGAAAGCTGCCTGTTCCAAAGGTCCTG
CACTTTGAACGGCATGATGGCTGGAGCAATCTGCTCATGAGTGAGGCCGATGGCGTCCTTTG
CTCGGAAGAGTATGAAGATGAACAAAGCCCTGAAAAGATTATCGAGCTGTATGCGGAGTGCA
TCAGGCTCTTTCACTCCATCGACATATCGGATTGTCCCTATACGAATAGCTTAGACAGCCGC
TTAGCCGAATTGGATTACTTACTGAATAACGATCTGGCCGATGTGGATTGCGAAAACTGGGA
AGAAGACACTCCATTTAAAGATCCGCGCGAGCTGTATGATTTTTTAAAGACGGAAAAGCCCG
AAGAGGAACTTGTCTTTTCCCACGGCGACCTGGGGGACAGCAACATCTTTGTGAAAGATGGC
AAAGTAAGTGGCTTTATTGATCTTGGGAGAAGCGGCAGGGCGGACAAGTGGTATGACATTGC
CTTCTGCGTCCGGTCGATCAGGGAGGATATCGGGGAAGAACAGTATGTCGAGCTATTTTTTG
ACTTACTGGGGATCAAGCCTGATTGGGAGAAAATAAAATACTATATTTTACTGGATGAATTG
TTTTAGtacctggagggaataATGAGTCTGAAAGAAAAAACACAATCTCTGTTTGCCAACGC
ATTTGGCTACCCTGCCACTCACACCATTCAGGCGCCTGGCCGCGTGAATTTGATTGGTGAAC
ACACCGACTACAACGACGGTTTCGTTCTGCCCTGCGCGATTGATTATCAAACCGTGATCAGT

TGTGCACCACGCGATGACCGTAAAGTTCGCGTGATGGCAGCCGATTATGAAAATCAGCTCGA
CGAGTTTTCCCTCGATGCGCCCATTGTCGCACATGAAAACTATCAATGGGCTAACTACGTTC
GTGGCGTGGTGAAACATCTGCAACTGCGTAACAACAGCTTCGGCGGCGTGGACATGGTGATC
AGCGGCAATGTGCCGCAGGGTGCCGGGTTAAGTTCTTCCGCTTCACTGGAAGTCGCGGTCGG
AACCGTATTGCAGCAGCTTTATCATCTGCCGCTGGACGGCGCACAAATCGCGCTTAACGGTC
AGGAAGCAGAAAACCAGTTTGTAGGCTGTAACTGCGGGATCATGGATCAGCTAATTTCCGCG
CTCGGCAAGAAAGATCATGCCTTGCTGATCGATTGCCGCTCACTGGGGACCAAAGCAGTTTC
CATGCCCAAAGGTGTGGCTGTCGTCATCATCAACAGTAACTTCAAACGTACCCTGGTTGGCA
GCGAATACAACACCCGTCGTGAACAGTGCGAAACCGGTGCGCGTTTCTTCCAGCAGCCAGCC
CTGCGTGATGTCACCATTGAAGAGTTCAACGCTGTTGCGCATGAACTGGACCCGATCGTGGC
AAAACGCGTGCGTCATATACTGACTGAAAACGCCCGCACCGTTGAAGCTGCCAGCGCGCTGG
AGCAAGGCGACCTGAAACGTATGGGCGAGTTGATGGCGGAGTCTCATGCCTCTATGCGCGAT
GATTTCGAAATCACCGTGCCGCAAATTGACACTCTGGTAGAAATCGTCAAAGCTGTGATTGG
CGACAAAGGTGGCGTACGCATGACCGGCGGCGGATTTGGCGGCTGTATCGTCGCGCTGATCC
CGGAAGAGCTGGTGCCTGCCGTACAGCAAGCTGTCGCTGAACAATATGAAGCAAAAACAGGT
ATTAAAGAGACTTTTTACGTTTGTAAACCATCACAAGGAGCAGGACAGTGCTGAacgaaact
c
