# Supplementary material for: Complete genome sequence of Helicobacter pylori B128 7.13 and a single‐step method for the generation of unmarked mutations
Source: Helicobacter. 2019 May 7;24(4):e12587. doi: 10.1111/hel.12587 (PMC6618122; doi:10.1111/hel.12587)
Supplement: Supplementary file 3 [file HEL-24-na-s003.docx]

**Table S1.** Comparison of the closed genome of B128 7.13 reported in this study with that of the B128 7.13 genomes reported in the study by Noto *et al.,* (24). Noto and colleagues reported the Illumina sequences of three B128 7.13 isolates, denoted in the table as Noto_1, Noto_2 and Noto_3. Although there are minor differences in the number of reported total genes, the number of coding genes differs significantly in our B128 7.13 genome (1584) with those found by Noto *et al.* (1375-1485).

| **Strain** | **Length** | **GC %** | **Total Genes** | **Coding Genes** | **Annotation** | **Status** |
| --- | --- | --- | --- | --- | --- | --- |
| **B128.7.13** | 1675441 | ~38.8 | 1625 | 1584 | PROKKA | Closed genome |
| **Noto_1** | 1674115 | ~38.8 | 1638 | 1485 | NCBI | Chromosome |
| **Noto_2** | 1674214 | ~38.8 | 1638 | 1485 | NCBI | Chromosome |
| **Noto_3** | 1674350 | ~38.8 | 1647 | 1375 | NCBI | Chromosome |
