# Supplementary material for: Complete genome sequence of Helicobacter pylori B128 7.13 and a single‐step method for the generation of unmarked mutations
Source: Helicobacter. 2019 May 7;24(4):e12587. doi: 10.1111/hel.12587 (PMC6618122; doi:10.1111/hel.12587)
Supplement: Supplementary file 4 [file HEL-24-na-s004.docx]

**Table S2.** Primers used in this study. Sequences highlighted in bold represent restriction enzyme sites.

| **Name** | **Sequence (5’-3’)** |
| --- | --- |
| CagA3’EcoRI_F | **GAATTC**CTTAAAGGATTAAGGAATATC |
| CagA3’OE_R | CACGCCCAAAGACTTTGGTAGCCATATGCGTTAGTGTCTGTCGGGATATTTTAAGGTG |
| CagA5’OE_F | CACCTTAAAATATCCCGACAGACACTAACGCATATGGCTACCAAAGTCTTTGGGCGTG |
| CagA5’BamHI_R | **GGATCC**CATTGTTTCTCCTTACTATACCTAGTTTC |
| NapA-2W1S_F | CGCTTCTTGATTGATAATATCC |
| NapA-2W1S_R | CTGAGTCTACCGCCCAGTTC |
| CagA-2W1S_F | CGCTACCCTTTGTAATCCTTG |
| 2W1S_R | GAAGCTTGGGGCGCTTTGGCTAATTGG |
| Hpylori16S_F | GGAGTACGGTCGCAAGATTAAA |
| Hpylori16S_R | CTAGCGGATTCTCTCAATGTCAA |
