# Supplementary material for: Complete genome sequence of Helicobacter pylori B128 7.13 and a single‐step method for the generation of unmarked mutations
Source: Helicobacter. 2019 May 7;24(4):e12587. doi: 10.1111/hel.12587 (PMC6618122; doi:10.1111/hel.12587)
Supplement: Supplementary file 5 [file HEL-24-na-s005.docx]

**Table S3.** Identities and locations of SNPs between the parental B128 7.13 strain and strains, C-2W, N-2W and CN-2W generated in this study.

| **Chromosome position** | **Reference base** | **Mutant base** | **Mutation Type** | **Amino Acid Substitution** | **Gene** | **C-2W** | **N-2W** | **CN-2W** |
| --- | --- | --- | --- | --- | --- | --- | --- | --- |
| 47025 | G | GC | Frame shift | K146Q | cheA_1 | n/a | n/a | GC |
| 82508 | T | TA | Non-coding region | n/a | n/a | TA | TA | TA |
| 83749 | T | TC | Frame shift | G160G | eda | TC | TC | n/a |
| 88921 | T | TG | Frame shift | G277G | glk | TG | TG | n/a |
| 176385 | A | AG | Frame shift | L110V | yajR_1 | AG | n/a | AG |
| 245454 | C | T | Nonsynonymous | E235K | HP713_00241 | T | T | T |
| 245458 | A | C | Nonsynonymous | N233K | HP713_00241 | C | C | C |
| 270041 | A | AG | Frame shift | R286A | nuoL_1 | AG | n/a | AG |
| 412729 | T | TC | Frame shift | G160G | cysP_2 | n/a | n/a | TC |
| 598321 | A | AC | Non-coding region | n/a | n/a | n/a | AC | n/a |
| 638795 | A | AG | Frame shift | V111G | HP713_00644 | n/a | AG | AG |
| 807381 | G | GC | Frame shift | G66G | mnmG_2 | n/a | n/a | GC |
| 879886 | G | A | Nonsynonymous | A12T | ftsH_1 | n/a | n/a | A |
| 905765 | A | AG | Frame shift | G121G | obg_1 | AG | AG | AG |
| 937119 | G | T | Premature stop codon | E301* | fliF | T | T | T |
| 955509 | C | T | Synonymous | L116 | tlyA | n/a | T | n/a |
| 1505043 | A | G | Non-coding region | n/a | n/a | G | G | G |
| 1520797 | C | T | Nonsynonymous | P401S | HP713_01483 | T | T | T |
| 1575524 | A | C | Nonsynonymous | V185G | gpsA | C | C | C |
