# Supplementary material for: Complete genome sequence of Helicobacter pylori B128 7.13 and a single‐step method for the generation of unmarked mutations
Source: Helicobacter. 2019 May 7;24(4):e12587. doi: 10.1111/hel.12587 (PMC6618122; doi:10.1111/hel.12587)
Supplement: Supplementary file 6 [file HEL-24-na-s006.docx]

**Table S4.** Comparison of current counter selectable methods used for the mutagenesis of *H. pylori.*

| **Study** | **Method** | **Reported efficiency of recombination to mutant allele** | **Advantages** | **Disadvantages** |
| --- | --- | --- | --- | --- |
| Copass *et al.,* 1997 | Generation of unmarked and scarless point mutations in strain G27 by positive selection for kanamycin resistance followed by sucrose-based counter-selection using the *sacB* cassette from *B. subtilis* | Not reported | Generated unmarked point mutations in the *vacA* gene. | 1) Two step transformation required. 2) Relatively high spontaneous resistance to sucrose has been reported in *H. pylori.* 3) Method only validated in strain G27. |
| Debowski *et al.,* 2012 | Generation of unmarked gene deletions in strains G27, X47 and 26695 by exploiting the difH/XerH recombination system in *H. pylori.* XerH-mediated excision of difH sequences flanking a rpsL-cat cassette enables selection for unmarked gene deletions based on chloramphenicol sensitivity and streptomycin resistance. | 100% | 1) Uses a single step transformation of mutagenic plasmid 2) Validated in three *H. pylori* strains | Cannot be used for the generation of point mutations or in-frame insertions due to the presence of a 40 base pair scar region at the target loci |
| Dailidiene *et al.,* 2006 | Generation of unmarked and scarless gene deletions by using a two gene cassette encoding the *Campylobacter jejuni rpsL* and *erm* genes, which confers dominant streptomycin susceptibility and selectable erythromycin resistance, respectively. | 68% | Unmarked gene deletions were generated in four strains of *H. pylori,* 26695, SS1, X47 and G27 using this method | Genomic DNA used to transfer mutations between strains could lead to unlinked secondary site mutations. |
| Zhang *et al.,* 2012 | Generation of unmarked and scarless gene deletions in strain 26695 by positive selection for chloramphenicol resistance followed by sucrose-based counter-selection using the *sacB* cassette from *B. subtilis* | Not reported | Generated unmarked and sequential gene deletions in the same strain. | 1) Relatively high spontaneous resistance to sucrose has been reported in *H. pylori*. 2) Two step transfromation required. |
| This study | Generation of unmarked and scarless mutations by positive selection for kanamycin or Apramycin resistance followed by 2-deoxygalactose-based counter-selection using the *galK* cassette from *E. coli* | 59% | 1) Generated markerless and scarless in-frame insertons, gene deletions and sequential mutations in the same strain. 2) Uses a single step transformation of mutagenic plasmid | Method only validated in B128 7.13 |
