# Supplementary material for: Complete genome sequence of Helicobacter pylori B128 7.13 and a single‐step method for the generation of unmarked mutations
Source: Helicobacter. 2019 May 7;24(4):e12587. doi: 10.1111/hel.12587 (PMC6618122; doi:10.1111/hel.12587)
Supplement: Supplementary file 7 [file HEL-24-na-s007.docx]

**Table S5.** Recombination efficiencies to mutant alleles in each strain generated in this study.

| **Strain** | **Number of 2-DOG resistant colonies tested** | **Number of colonies positive for mutant allele by PCR** | **Recombination efficiency to mutant allele (%)** | **Average recombination frequency (%)** |
| --- | --- | --- | --- | --- |
| C-2W | 18 | 8 | 44 | 59 |
| N-2W | 15 | 10 | 67 |  |
| CN-2W | 7 | 4 | 57 |  |
| Δ*cagA* | 9 | 6 | 67 |  |
